# Weed Functional Diversity as Affected by Agroecological Service Crops and No-Till in a Mediterranean Organic Vegetable System

**DOI:** 10.3390/plants9060689

**Published:** 2020-05-28

**Authors:** Corrado Ciaccia, Laura Armengot Martinez, Elena Testani, Fabrizio Leteo, Gabriele Campanelli, Alessandra Trinchera

**Affiliations:** 1 Council for Agricultural Research and Economics (CREA), Research Centre for Agriculture and Environment, Via della Navicella, 2, 00184 Roma, Italy; elena.testani@crea.gov.it (E.T.); alessandra.trinchera@crea.gov.it (A.T.); 2 Research Institute of Organic Agriculture (FiBL), Ackerstrasse 113, Postfach 219, 5070 Frick, Switzerland; laura.armengot@fibl.org; 3 Council for Agricultural Research and Economics (CREA), Research Centre for Vegetable and Ornamental Crops, Via Salaria 1, 63030 Monsampolo del Tronto, Italy; fabrizio.leteo@crea.gov.it (F.L.); gabriele.campanelli@crea.gov.it (G.C.)

**Keywords:** agroecology, weed biodiversity, mulch, community composition, ecological weed management

## Abstract

This paper explores the effect of agroecological service crops (ASCs), i.e., crops included in the crop rotation for their ecosystem services, terminated with an in-line tillage roller crimper (ILRC) on weed community composition and their functional traits in comparison to a tilled control without ASC. A two-year study was performed in a long-term experiment with vegetables under organic management. Four different cereal crops were introduced as ASCs. Weed abundance and richness and the functional traits were assessed at three different stages, i.e., before and after ASC termination and before harvest of the following crop, melon. All the ASCs showed strong weed suppression, with few differences between the cereals tested. Weed communities with ASCs had later flowering onset and wider flowering span compared to the control, which positively affects weed dispersal and attraction of beneficial insects. However, weed communities with ASCs had higher values for traits related to competition (specific leaf area, seed weight and more perennials). A trade-off between weed suppression and selection of more competitive weed communities by the introduction of ASCs managed with the ILRC should be evaluated in the long-run. The use of the ILRC alternating with other soil management practices seems the more effective strategy to benefit from the minimal soil tillage while avoiding the selection of disservice-related traits in the weed community.

## 1. Introduction

The implementation of agroecological practices can improve the sustainability of production systems, minimizing the negative environmental impacts of agriculture and increasing the ecological services provided besides the yield [1,2,3].

The introduction of agroecological service crops (ASCs) in cropping systems (i.e., crops planted to obtain ecosystem services more than strictly focused on yield improvement, such as catch crops, cover crops, trap crops or, green manures) can provide or promote agroecosystem services by modifying the associated diversity, like that of spontaneous flora [4]. This can happen regardless of the placement of the ASCs in the crop rotation (e.g., as break crops—between two cash crops—or living mulch—intercropped with cash crop) and of the termination methods [5]. The ecological services provided by ASC depend on the botanical family, species or mixtures chosen [6]. The ASCs occupy the ecological niches otherwise occupied by weeds and therefore they represent a key strategy for weed management in organic systems, where the use of herbicides is not allowed [7,8]. Including cereals as ASCs can contribute to weed management due to their strong competitive and allelopathic effect compared to other crop families [7].

Reducing soil tillage can influence weed communities, for instance by selecting less competitive species [9]. Reduced tillage can benefit soil fertility (e.g., reduced soil erosion and organic matter loss), pest management (e.g., increasing biodiversity), and climate change mitigation (e.g., carbon sequestration, reduced energy consumption and emissions of greenhouse gases) [9,10,11,12], but generally increases weed infestation [13,14]. In this regard, several authors have pointed out that weed control effectiveness can be strongly improved by combining reduced or no tillage with ASCs [7,15,16]. ASC termination creates an organic mulch on the soil surface (i.e., mowing or flattening) that can function as a physical barrier to incoming light, weed emergence and seedlings development, therefore selecting species more suited to the modified soil microenvironment. Surface residues decrease the average soil temperature and the daily fluctuations, reducing germination rates of many weed species [13]. Furthermore, reduced tillage can change the functional attributes of the weed community, acting as selective driver [7,9,13,15,17].

In vegetable production systems, where crops are often transplanted and not sowed, the no-till approach has been recently introduced with the in-line tillage/roller crimper (ILRC) [16,18,19]. A vertical disk and a chisel installed in-line on a roller allow to till a narrow area (0.2–0.3 cm), while the remaining area is not tilled, and it is covered by the previously flattened ASC. The ILRC crushes the vascular tissue of ASCs without completely cutting the stems. In this way, the residue surface in close contact with soil microorganisms is reduced, slowing down degradation and assuring weed suppression over time [20]. In particular, Poaceae species introduced as ASCs are considered particularly suitable for weed suppression with ILRC termination, thanks to the high amount of biomass produced and to the slow decay of their residues due to high C:N ratio [21,22].

Several authors pointed out the importance of the correct amount of ASC biomass production for an effective weed management [16,18,23,24], but very few studies have focused on the effect of the ASCs and their related management on changes in weed community composition and functional traits [18,25]. Species occurrence relies on a suite of response ‘traits’ able to guarantee their survival in a given ecosystem [26] and it is mainly related to strategies to overcome weed control practices in agroecosystems. Each species is characterized by several functional traits that can be related to production (e.g., attraction of beneficials, hosting mychorriza) or to non-production agroecosystem services (e.g., bird or arthropod population [26,27]).Moreover, the potential additive effect of ASC mixture on weed suppression has been reported [23] but, to the best of our knowledge, no work has focused on the effect of different ASC species of the same botanical family.

This study aims to compare the effect of the introduction in the crop rotation of different cereal ASCs and their associated management, i.e., use of the ILRC and no till, with a bare soil (ASC no, fallow and tilled system) on weed abundance, richness and functional traits at three different crop stages: termination of the ASCs, the initial stage of the growing period of the following cash crop (melon, *Cucumis melo* L.) and harvest. The study was performed over 2 years in a long-term experiment with vegetables under organic farming.

We hypothesise that, before termination: (i) the ASC introduction reduces weed abundance and species richness compared to the ASC no; (ii) the ASC introduction affects weed communities by selecting species adapted to the modified micro-climatic and edaphic conditions and/or differing in specific functional traits related to main services (e.g., attraction of beneficials) and disservices (e.g., competitiveness); after termination: (iii) the ASC mulch may suppress weed emergence and growth during the cash crop cycle, i.e., in melon, counteracting the potential negative effects of the no-tillage on weed abundance; (iv) the ASC mulch can represent a selective filter on weed traits; and (v) even ASC species from the same family affect weed development and traits differently due to different interference ability in the growing season and effect of their residues decay after termination.

## 2. Results

### 2.1. Effect of ASC Introduction on Weed Density/Cover, Species Richness, Community Composition and Functional Traits

Overall, the introduction of ASCs compared to a tilled fallow control affected all the traits assessed. Year and the year x ASC interaction are significant for several traits (Table 1). Since the factor year includes the effects of weather conditions but also a different seedbank (close but different fields of the same rotation system, as reported in Material and Methods), we performed the analyses separately for each experimental year in order to better disentangle and observe common/diverging trends of the effect of the ASCs (Table 2).

At the termination stage (TE), weed communities growing in the presence of ASCs (hereafter, ASC yes) had more creeping species (CRP), higher specific leaf area (SLA) and duration of flowering (DFF) and earlier flowering onset (less BFF) compared with ASC no (hereafter, ASC no) in both years, while they had less affinity for light (L) in 2014 and the highest affinity for temperature (T) in 2015 (Table 2). No significant differences are observed for the remaining traits. At the plant growing stage (PG) traits of weed communities in ASC yes compared to ASC no had the highest values of seed weight (SWT), SLA, DFF and the lowest affinity to temperature (T) and soil nutrient condition (N) in both the years, while they had the highest values of seed bank longevity (SBL) and canopy height (CH) and lower of BFF in 2014 only. At the end of harvest (EH), similar trends to those at the PG phase are observed, except for higher values of perennials (PRN), L, T and species richness (R) in 2014, higher value of DFF in 2015 and lower R in 2014, in ASC yes compared to the ASC no.

**Figure 1:**
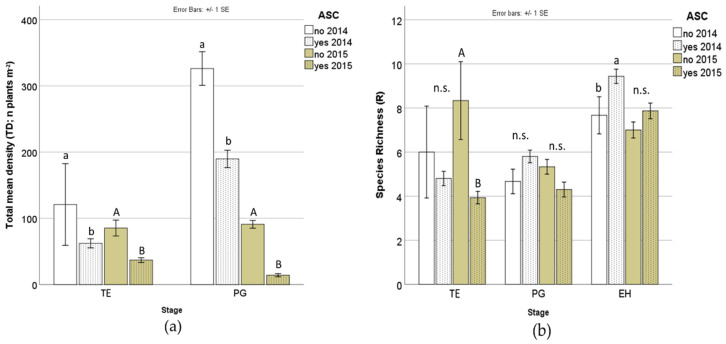
Weed total density (**a**) and species richness (**b**) as affected by ASC and Year factors at different plant growing stages. *Different Roman letters indicate significant different weight according to lsmeans; n.s.: not significant. ASC: agroecological service crop; TE: termination; PG: plant growth; EH: end of harvest.*

In both years, weed density (TD) was significantly lower in ASC yes than in the ASC no at TE and PG (Table 2, Figure 1a), whereas weed cover (C) showed significant differences only in 2015 (32.8% vs. 83.3% in ASC yes and ASC no, respectively; 87.5% vs. 80.8% in 2014). On the other hand, species richness did not significantly differ between ASC yes and ASC no, with the exception of the TE stage in 2015, where ASC no recorded the highest R value, and at EH stage in 2014, where R was higher in ASC yes than ASC no (Table 2, Figure 1b).

**Figure 2:**
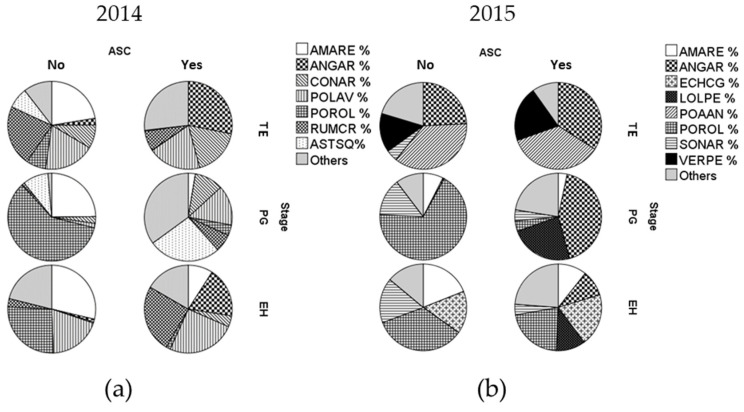
Weed species relative density at each stage in ASC no (No) and ASC yes (Yes) in 2014 (**a**) and in 2015 (**b**). ASC: agroecological service crop; TE: termination; PG: plant growth; EH: end of harvest; ‘No’ refers to the ASC no, i.e., fallow plots, without ASCs and ‘Yes’ to ASC yes, i.e., presence of ASCs. AMARE: *Amaranthus retroflexus* L.; ANGAR: *Anagalllis arvensis* L.; CONAR: *Convolvulus arvensis* L.; ECHCG: *Echinochloa crus-galli* (L.) P.Beauv.; LOLPE: *Lolium perenne* L.; POAAN: *Poa annua* L.; POLAV: *Polygonum aviculare* L.; POROL: *Portulaca oleracea* L.; RUMCR: *Rumex crispus* L.; SONAR: *Sonchus arvensis* L.; ASTSQ: *Symphyotrichum squamatum* (Sprengel) Nesom; VERPE: *Veronica persica* Poir.

The weed community composition in terms of relative densities of main species for the two experimental years is reported in Figure 2. Results show differences between years and in relation to ASC. In 2014, at TE the ASC yes system showed the dominance of four species, namely scarlet pimperlane (*Anagallis arvensis* L., ANGAR), field bindweed (*Convolvulus arvensis* L., CONAR), prostrate knotweed (*Polygonum aviculare* L., POLAV) and curly dock (*Rumex crispus* L., RUMCR). On the other hand, minor species (i.e., others, Figure 2) represented more than 25% of the total individuals in the community, whereas their relevance was reduced in fallow, where redroot pigweed (*Amaranthus retroflexus* L., AMARE), RUMCR and POLAV showed the highest relative density. In both years, at PG weed community is characterized by the dominance of common porcelain (*Portulaca oleracea* L., POROL), representing over 60% of the total in fallow. On the other hand, ASC yes had a more balanced community at PG and EH stages, with lower dominance of species than in ASC no plots. AMARE represented about 25% of the total for all the stages in 2014 ASC no, whereas ANGAR characterized weed community in 2015 ASC yes.

### 2.2. Effect of ASC Species on Weed Density/Cover, Species Richness and Community Functional Traits

None of the studied traits and parameters show significant difference among the ASC species at TE and EH, whereas at PG ASC species show an effect on CH, SLA, T, R and TD (Table 3). Year factor, alone or in interaction with the ASC species is significant for most of the studied traits and parameters at TE and PG stage, as far as on five out 13 at EH. Statistical analyses were then performed within each experimental year (Table 4).

At TE, differences in the traits of weed communities growing with different ASC are observed only for SLA (Table 4). Results for the differences among ASC species are reported in Figure S2. Barley selected a weed community characterized by the lowest SLA, whereas spelt the one with the highest value. At PG, the ASC species affected CH, SLA, BFF, DFF, T due to the different performance of the rye compared with the rest of ASCs in 2015. Rye selected a weed community with the highest CH and BFF as well as the lowest SLA and DFF. No significant differences are observed in the EH for the selected traits.

**Figure 3:**
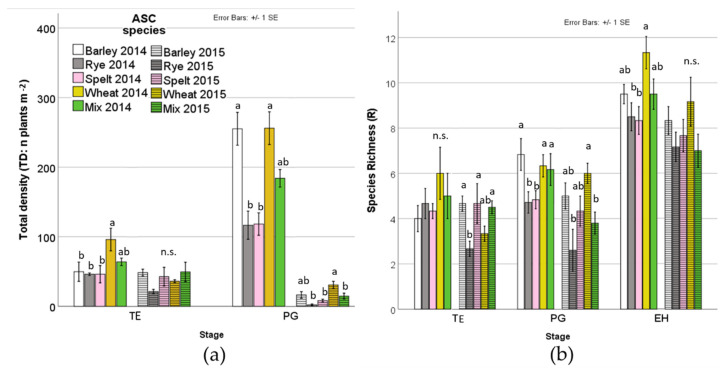
Weed total density (**a**) and species richness (**b**) as affected by ASC species over the two years experiment. ASC: agroecological service crop; TE: termination; PG: plant growth; EH: end of harvest. *Different Roman letters indicate significant different weight according to lsmeans and Tukey post-hoc tests; n.s.: not significant.*

The ASC species influenced the total density at TE in 2014 and PG in both years. Wheat shows the highest weed density in 2014, whereas rye and spelt shows the lowest value, together with mix, at PG both years (Table 4, Figure 3a). No significant differences among ASC species in weed cover are observed at EH ranging between 75.0% (spelt) and 87.5% (wheat) in 2014 and between 18.8% (mix) and 46.7% (barley) in 2015 (data not shown). The ASC species influenced species richness at the TE in 2015, at PG in both years and at EH in 2014 (Table 4, Figure 3b). Rye shows the lowest species richness together with wheat at TE in 2015, with spelt in 2014 and with mix in 2015 at PG and with spelt at EH in 2014 (Figure 3b).

## 3. Discussion

These results evidence the strong effect of the year on the weed community structure. On the one hand, it can be related to potential different seedbanks between the two fields in the two experimental years, even if part of the same long term trials, as suggested by differences in main species between weed communities in the two experimental years (Figure 2). On the other hand, the different climatic conditions that occurred in the two years may have strongly influenced the results, since differences were recorded also in traits distribution (Table 1 and Table 3). In particular, heavy rainfalls were recorded during both the ASC and the melon crop cycles in 2014 (1166 mm between November and July vs. 452 mm historically recorded for the same period), with peaks of air temperature up to 3.6 °C higher than the mean value that occurred in January–February, and 1.9 °C lower than the mean long-term trend during melon cycle (May–July). The year 2015 was instead characterized by an average temperature up to 3.2 °C above the mean value during the melon cycle (data not shown). Heavy rainfall in 2014 could have driven towards higher weed density cover at EH than in 2015.

### 3.1. Effect of ASC Introduction on Weed Density/Cover, Species Richness and Community Composition and Functional Traits

**ASCs termination**—At TE, weed community composition analysis provides information about the effect of ASC on weed control and on the selection of traits, potentially influencing the seedbank for the following crops in rotation and particularly for the subsequent winter ones. In agreement with our first hypothesis, our results confirm the role of ASC in reducing weed abundance through direct competition, as suggested by the significant reduction of weed density compared to ASC no [28]. However, the ASCs do not seem to necessarily reduce weed community richness, which positively contributes to the conservation of a diverse weed community. The results at TE also confirmed our second hypothesis: in particular, the selection of CRP trait in ASC yes community can be explained by the need to develop through the dense biomass of the cereals to capture the light. In accordance with previous studies [15], changes in Ellenberg climatic indicators L (2014) and T (2015) suggest that ASCs are able to modify the environment by selecting species with lower light and higher temperature affinity than in fallow (Table 2).

The selection of weed species with wider DFF and earlier BFF in ASC yes compared to fallow at TE and in the following stages analysed provides twofold information. On one hand since the ASC cycle occurs between November and April, an early BFF indicates an early reproductive stage able to influence weed spreading, on the other hand a wider DFF may determine attraction of potential beneficial insects for a longer period [29,30]. However, the reduction of weed density in ASC plots counteracts the risk of weed spreading (Figure 1a).

**Plant growing stage**—The results at PG provide information about the role of the management (i.e., tillage vs. no tillage and ASC mulch absence vs. presence) on weeds in the first critical crop phases. The results on total weed density demonstrate the ability of the mulch to control weeds, confirming our third hypothesis, in accordance with previous studies [7,15,18,19]. Interestingly, the mulch did not markedly reduce species richness. Results at this stage also confirm our fourth hypothesis. The presence of the mulch clearly contributes to modify the soil microenvironment, leading to selection of species with low T and N Ellenberg scores (Table 2). Low T scores in ASC yes are in line with the results of Canali et al. [16], who reported a 2–3 °C degree reduction of soil temperature in roller crimper treatments relative to the tilled control without mulch. Moreover, according to Reberg-Horton et al. [31] cereal mulches may influence weed dynamics by immobilizing N, which may favour species with an oligotrophic attitude [19,32]. Armengot et al. [9] obtained similar result for reduced tillage conditions compared to conventional management.

In contrast to what was seen in the TE stage, no selection of creeping species was observed during the plant growing stage. Our findings on the traits related to disservices (competition with crop) are not in agreement with previously reported results. For instance, perennial species did not increase, in contrast with the results of other literature studies conducted on no till systems [9], and the acknowledged relationship between seed weight and their persistence in the seedbank is not observed. However, Armengot et al. [9] have highlighted a similar result by analysing the effects of the tillage system on the weed communities from seven field trials in different climatic regions across Europe. The selection of a weed community with lower persistence of the seedbank (SBL), as in 2014 ASC yes systems, leads to a potential reduction of weeds over time when the system is properly managed with practices aimed at reducing the weed seed spreading. This result was not confirmed in 2015, indicating that the dynamics of this seedbank-related trait induced by the ILRC cannot be easily observed in the short-term. On the contrary, the higher seed weight in ASC yes in both years could be related to lower light availability under the mulch, as small-seeded species are initially more dependent on light compared to large-seeded ones. Furthermore, the heaviest seeds take advantage in terms of seedling survival and success of emerging under competitive environment [13,33,34]. Our results do not show differences in affinity for light between weed communities at this stage. The high values of the SLA trait in ASC yes systems, which relates to the potential of a plant to respond to a change in stress exposure, evidence a potential risk of selecting a competitive flora by the ILRC in the long run [19]. This result suggests that soil management strategies (e.g., to alternate flattening and green manure) should be diversified in order to avoid the potential selection of competitive flora in the long run, yet exploiting the weed suppression effect well demonstrated by the ILRC technology. Regarding the phenological traits, the compared systems affect both flowering onset (in 2014) and span, potentially influencing other taxa that depend on flower availability. The choice of weed management strategies towards the maintenance of such weed community diversity can therefore be an opportunity to positively benefit the agroecosystem by favouring fitness, survival and reproduction of beneficial insect populations, which are directly dependent on flowers availability [6,29]. Further research is required to disentangle the potential effect of attracting pollinators and other beneficials related to phenological response traits.

**End of harvest stage**—Similar results on weed community traits between PG and EH highlighted only slight changes of the weed communities during the crop cycle, as similar frequencies of the main species in both the stages were recorded (Figure 2). This result indicates that the mulch maintains both a filter action on weed traits throughout the crop cycle [35] and the ability to suppress weeds until the end of the crop cycle [7,16,17,18,19,20] without reducing weed richness. Finally, the lower L Ellenberg score, found in the weed communities of ASC yes treatments in 2014, suggests a potential effect of the mulch in favouring the emergence of sciaphilous species during the crop cycle [15]. The difference in the SWT trait supports this finding, since larger seed species are initially less dependent on light [13,33].

### 3.2. Effect of ASC Species on Weed Density/Cover, Species Richness and Community Composition and Functional Traits

Few differences of weed communities’ traits among the ASCs species during their growing season were recorded, due to the similarity between the tested cereals in habitus, phenology and biomass production at the different stages. This result confirms the role of grass species as ASC in weed management, due to the slow decay of the residues and, consequently, of the mulch, in the first critical phases of the vegetable crop cycles. However, rye shows a general trend of total weed density reduction, probably due to its acknowledged allelopathic activity [36,37,38]. The role of the ASC species is more evident after termination, when the mulches differently influence weed communities’ structural parameters, thus partially confirming our final hypothesis. The higher weed suppression ability of rye residues, particularly in 2014 when weather conditions were more favourable to weed development, is probably due to its allelopathic potential. However, the degradation of allelochemicals initially released by the mulch probably led to similar results in weed cover to the other ASC species at EH in both years. Rye shows an effect also in trait selection, driving into a community with the highest competitive ability (the highest canopy height) and resource use ability (SLA). This result is in accordance to the LHS model [33,39] that could be interpreted as a response of the weed community to allelopathic stress conditions. Similarly, the changes in flowering parameters in rye suggest that the choice of ASC species can also be made based on the potential benefit deriving from the attractiveness of the flora selected by the ASC species themselves.

## 4. Materials and Methods

### 4.1. Site Characterization

A two-year field experiment was carried out in 2013–2014 and 2014–2015 at the MOnsampolo VEgetables organic Long-Term Experiment (MOVE LTE) of the Research Centre for Vegetable and Ornamental Crops of the Council for Agricultural Research and Economics (CREA-OF), located in Monsampolo del Tronto (AP) (latitude 42°53′ N, 13°48′ E), in the coastal area of the Marche Region, Central Italy. The MOVE LTE, established in 2001, consists in a 4-year crop rotation with 6 main crops and 3 ASCs (break crops). Additional information about the MOVE LTE are available in Campanelli et al. [40]. The site is characterized by a “thermomediterranean” climate [41], with an average total annual precipitation of 564 mm and average temperatures of 9 and 20 °C in October–March and April–September periods, respectively. According to Soil Taxonomy of the U.S. Department of Agriculture [42], at the field trial site the soil was a Typic Calcixerepts fine-loamy, mixed thermic one.

### 4.2. Experiment Setup and Treatments

The experiment was divided on two crop cycles, the ASC and the cash crop (the melon) ones, corresponding to two different layouts. During the ASC cycle, the experimental design was a randomized complete block design (RCBD) with 3 replications (blocks) and one factor, assigned to the ASC treatment. Six ASC treatments were compared: (i) wheat (*Triticum aestivum* L.); (ii) barley (*Hordeum vulgare* L.); (iii) rye (*Secale cereale* L.); (iv) spelt (*Triticum dicoccum* L.); (v) mix (made up of all ASC seeds equally divided by weight); and (vi) tilled fallow as control (ASC no). At the end of ASC cycle, the experimental layout was modified in a split-plot design with the presence/absence of the cash crop assigned to the sub-plot factor. In particular, each main plot (27.0 m^2^) was in turn split in three equal subplots (9.0 m^2^) to obtain a split-plot design for the melon cycle, where in two subplots the melon was transplanted (melon yes, one with hand weeding management during melon cycle, and the second unweeded) and one, where it was not (no melon, unweeded). The present study only took into consideration the unweeded subplots (unweeded melon yes and no melon) to further test the effect of the crop on weed community. Preliminary analyses of the data showed that the melon did not have any effect on weed parameters in both years. Data were therefore pooled across the unweeded melon yes/no treatments. Since the field experiment was part of the 4-year rotation of the MOVE LTE, the spatial position of the plots was different in the two years. Accordingly, the year factor includes effects potentially attributable both to weather conditions and seedbank, although the fields in the two years were close and part of the same rotation. Accordingly, our study only accounts for the short-term effects of the presence and the related management of the ASCs (no cumulative effects of the two years). No weeding was performed during the ASC cycles also. ASC were sown after tomato crop (*Solanum lycopersicum*, L.) at a rate of 250 kg ha^−1^ on 31st October and on 05th November in 2013 and 2014, respectively, in each plot (18 m^2^). Before 2014 and 2015 sowing, seed germinability of ASCs was tested, resulting about 90%. On 29th and 30th April in 2014 and 2015, respectively, ASCs were terminated at full flowering stage by flattening. The machinery utilized was the in-line tillage roller crimper (ILRC) technology, which allowed to simultaneously prepare the melon transplanting furrows guaranteeing no till in the inter-row spaces [16]. In the fallow plots (ASC no), soil was ploughed to a depth of 20 cm at the end of October 2013 and 2014. Before melon transplanting, control plots were tilled with a rotary tiller (DL 2500; Maschio SPA, Padua, Italy) at a 15-cm depth, contemporary to ASC termination.

Melon (HF1 Anish) seedlings were 25 days old and were hand-transplanted at an inter-row × row distance spacing of 1.0 × 1.0 m (1.0 plant m^−2^) on 30th and 18th May in 2014 and 2015, respectively. In 2014, the melon harvest started on the 1st and was completed on the 13th of August, with a cropping cycle of 75 days. In 2015, the harvest started on 24 of July and terminated on 10th of August, with a cropping cycle of 84 days. The melon crop was irrigated with 1200 m^3^ ha^−1^ in 2014 and 1400 m^3^ ha^−1^ in 2015. At melon transplanting, the trial was fertilized using off-farm fertilizers allowed in organic farming according to the European regulation in force, corresponding to 50 kg ha^−1^ of N. An additional 10 and 8 kg ha^−1^ of N and K_2_O, respectively, were distributed by fertigation along the cropping cycle.

### 4.3. Measurements

Weed community composition was evaluated at ASC termination (TE), at plant (melon) growing (PG: 27 and 34 days after transplanting, DAT, for 2014 and 2015, respectively) and end of harvest (EH: 69 and 78 DAT for 2014 and 2015, respectively) stages. At each sampling stage, the weed density (at TE and PG) or cover (at EH)—both total (to provide Total Density, TD, and total Cover, C) and by-species—were recorded by placing three randomly-selected 0.25 × 0.25 m^2^ and 1.0 × 1.0 m^2^ quadrats within each plot for density and cover assessment, respectively. The weed cover abundance/dominance index was estimated according to the Braun–Blanquet scale, as modified by Pignatti [43]. Each Braun–Blanquet class was then converted to its midpoint cover value according to Wikum and Shanholtzer [44]. By-species density and cover provided measures of weed species richness (R).

### 4.4. Selection of Traits

Plant traits were selected from the weed functional traits database published by Bàrberi et al. [26] and from the literature The analysed traits were: growth form (CRP, creeping vs. not creeping species), life form (PRN, annual vs. perennials), seed bank longevity (SBL, short-term, i.e., equal to/shorter than 5 years; long-term, i.e., longer than 5 years), specific leaf area (SLA, mm^2^ mg^−1^), seed weight (SWT, mg), canopy height (CH, m, maximum height at maturity), beginning of flowering (BFF, month of the first flowering), flowering span (DFF, duration of flowering in months), and soil nutrient condition, temperature and light affinities (N, T and L, Ellenberg and Pignatti values).

The CRP was used to evaluate the selection by the ILRC of creeping/prostrate species (ability to escape termination [19] and to easily develop through the live mulch). The PRN (ability of the plants to grow during one or more seasons) and SBL (the ability of the seeds for remaining viable in the soil) provide information about the persistence over time. The competitiveness and the disservice related are evaluated through the SLA (higher values mean higher metabolic activity, often characterizing ruderal or competitor species [45]), SWT (the capacity to emerge from depth and colonize at a distance) and CH at maturity (competition for light with neighbouring plants) as effect traits, according to leaf–height–seed strategy scheme proposed by Westoby [39]. Both BFF and DFF are considered response traits (adaptation and persistence to environmental and management condition; [26]), but also providing service attracting pollinators (effect traits). The Ellenberg ecological indicator values, as modified by Pignatti [46], are not strictly traits but may serve as surrogates for corresponding stress tolerance traits.

### 4.5. Statistical Analysis

Community-level weighted mean of trait values (CWM), which measures the weighted average of traits for the species pool in the weed community, was calculated for each single trait using package “FD” [47] for R [48]. This community-aggregated metric represents the expected functional trait value of a random community sample, often representing the dominant trait value in a community. To homogenize the data, the trait weight across taxa CH, SWT and SLA were log-transformed, whereas BFF was linearized by angular transformation. For categorical traits, only one of the levels was analysed, i.e., perennial weeds CWM was analysed for PRN, the CWM of creeping for the CRP and the CWM of the long-term seeds for the SBL.

First, the effect of the ASC factor (ASC yes/ASC no) on the CWM of each trait and on species richness and density/cover was analysed through linear mixed-effect models, i.e., all the different ASCs were poled and compared with the ASC no. ASC, Year (2013/14 vs. 2014/15, hereafter reported as 2014 and 2015, respectively) and their interaction were included as fixed factors. Block was included as a random factor for the TE data and plot nested to block for the PG and EH and for the data on species richness and density/cover, since more than one datapoint per plot was included. The effect of the ASC species (without including the ASC no plots) on the same variables was also tested. Data were transformed, when necessary, to meet the normality and homoscedasticity requirements. All the analyses were performed in R 3.5.1 (R Development Core Team, 2014) with the “lme4” package [49] for mixed models, “lmerTest” to calculate the confidence intervals and the significance of the effects [50] and “lsmeans” for the post-hoc test of the different variables [51].

## 5. Conclusions

The study results point out the role of ASCs on weed control and community composition before and after the termination by the ILRC. In particular, the ASC mulch counteracted the potential promotion of weeds due to the lack of tillage and acted as a filter for the community structure without significantly reducing species richness. Similar effects on weed communities and traits were obtained by all the cereals studied, due to their similar phenotypic characteristics and growth habit.

The analysis of the weed community during the ASC cycle can provide useful information about the selection of weed traits and, potentially, of the seedbank for the subsequent crop in the rotation. This approach could drive choices for weed management strategies based on the foreseen weed community structure. In this context, it seems beneficial to integrate the ILRC in a suite of complementary soil practices to reduce a potential selective pressure on weeds and the establishment of a new flora, increasingly competitive with the cash crops. However, the ILRC technique is generally applied on ASCs sowed after soil tillage, reducing the effect of repeated no till on the weed selection. Moreover, ILRCs have proved to be an effective strategy to introduce the no tillage also in vegetable rotation, benefiting from the reduced soil disturbance while maintaining a low weed infestation. Long-term studies considering ILRC should be carried out in order to highlight the selection ability of mulch-based no till practice in designing weed community assembly trajectory.

## Figures and Tables

**Table 1:** Results of linear mixed model for tested parameters in the two-years experiment as affected by the year, and the ASC factors.

		CRP	PRN	SBL	SWT	CH	SLA	BFF	DFF	L	T	N	R	TD/C
**ASC**														
**TE**	Sig.	***	n.s.	n.s.	n.s.	n.s.	*	*	**	**	n.s.	n.s.	**	*
**PG**	Sig.	n.s.	*	***	***	n.s.	***	***	***	*	***	***	n.s.	***
**EH**	Sig.	n.s.	n.s.	n.s.	**	***	n.s.	***	n.s.	n.s.	n.s.	**	*	n.s.
**Year**														
**TE**	Sig.	***	n.s.	n.s.	***	**	n.s.	*	***	**	***	*	n.s.	n.s.
**PG**	Sig.	n.s.	n.s.	n.s.	n.s.	n.s.	n.s.	n.s.	n.s.	n.s.	***	n.s.	n.s.	***
**EH**	Sig.	n.s.	n.s.	n.s.	**	***	n.s.	***	n.s.	n.s.	n.s.	**	*	n.s.
**Year x ASC**														
**TE**	Sig.	**	n.s.	n.s.	n.s.	n.s.	**	n.s.	***	***	**	**	*	n.s.
**PG**	Sig.	n.s.	n.s.	**	n.s.	n.s.	n.s.	*	n.s.	n.s.	n.s.	n.s.	*	n.s.
**EH**	Sig.	n.s.	***	n.s.	n.s.	n.s.	n.s.	n.s.	n.s.	n.s.	*	n.s.	n.s.	n.s.

ASC: agroecological service crop; TE: termination; PG: plant growth; EH: end of harvest; CRP: creeping vs. not creeping species; PRN: perennial life form vs. annual; SBL: long-term seed bank longevity vs. short-term; SWT: seed weight; CH: canopy height; SLA: specific leaf area; BFF: beginning of flowering (flowering onset); DFF: duration of flowering (flowering span); L: light affinity; T: temperature; N: soil nutrient condition (N, T and L, Ellenberg and Pignatti values); R: species richness; TD/C: total density for TE and PG stages and total cover for EH one. Probability level: n.s., not significant; *, *p* ≤ 0.05; **, *p* ≤ 0.01; ***, *p* ≤ 0.001.

**Table 2:** Weed traits, Ellenberg ecological indicators, species richness and total density/cover of the weed communities in the two-years experiment as affected by the ASC factor.

	CRP	PRN	SBL	SWT	CH	SLA	BFF	DFF	L	T	N	R	TD/C
**TE 2014**	N < Y	n.s.	n.s.	n.s.	n.s.	N < Y	N > Y	N < Y	N > Y	n.s.	n.s.	n.s.	N > Y
**TE 2015**	N < Y	n.s.	n.s.	n.s.	n.s.	N < Y	N > Y	N < Y	n.s.	N < Y	n.s.	N > Y	N > Y
**PG 2014**	n.s.	n.s.	N > Y	N < Y	N < Y	N < Y	N > Y	N < Y	n.s.	N > Y	N > Y	n.s.	N > Y
**PG 2015**	n.s.	n.s	n.s	N < Y	n.s.	N < Y	n.s.	N < Y	n.s.	N > Y	N > Y	n.s.	N > Y
**EH 2014**	n.s.	N < Y	N > Y	N < Y	N < Y	N < Y	N > Y	n.s.	N > Y	N > Y	N > Y	N < Y	n.s.
**EH 2015**	n.s.	n.s.	n.s.	N < Y	n.s.	N < Y	n.s.	N < Y	n.s.	n.s.	n.s.	n.s.	N > Y

ASC: agroecological service crop; TE: termination; PG: plant growth; EH: end of harvest. ^2^ ‘N’ refers to the ASC no, i.e., fallow plots, without ASCs and ‘Y’ to ASC yes, i.e., presence of ASCs. ^3^ CRP: creeping vs. not creeping species; PRN: perennial life form vs. annual; SBL: long-term seed bank longevity vs. short-term; SWT: seed weight; SLA: specific leaf area; BFF: beginning of flowering (flowering onset); DFF: duration of flowering (flowering span); L: light affinity; T: temperature; N: soil nutrient condition (N, T and L, Ellenberg and Pignatti values); R: species richness; TD/C: total density for T and PG stages and total cover for EH. Probability level: n.s., not significant; N < Y, significantly higher values in the presence of ASCs, N > Y significantly lower values in the presence of ASCs.

**Table 3:** Results of linear mixed model in the two-years experiment as affected by the Year and the ASC species.

		CRP	PRN	SBL	SWT	CH	SLA	BFF	DFF	L	T	N	R	TD/C
**ASC species**														
**TE**	Sig.	n.s.	n.s.	n.s.	n.s.	n.s.	n.s.	n.s.	n.s.	n.s.	n.s.	n.s.	n.s.	n.s.
**PG**	Sig.	n.s.	n.s.	n.s.	n.s.	***	n.s.	n.s.	**	n.s.	**	n.s.	*	***
**EH**	Sig.	n.s.	n.s.	n.s.	n.s.	n.s.	n.s.	n.s.	n.s.	n.s.	n.s.	n.s.	n.s.	n.s.
**Year**														
**TE**	Sig.	***	n.s.	**	***	**	*	n.s.	n.s.	***	***	***	*	***
**PG**	Sig.	n.s.	n.s.	n.s.	n.s.	n.s.	n.s.	n.s.	n.s.	n.s.	***	n.s.	n.s.	***
**EH**	Sig.	n.s.	n.s.	n.s.	**	***	n.s.	***	n.s.	n.s.	n.s.	**	*	*
**Year x ASC sp.**														
**TE**	Sig.	n.s.	n.s.	n.s.	n.s.	n.s.	n.s.	n.s.	n.s.	n.s.	n.s.	n.s.	n.s.	*
**PG**	Sig.	*	n.s.	n.s.	n.s.	***	**	**	**	n.s.	**	n.s.	n.s.	**
**EH**	Sig.	n.s.	n.s.	n.s.	n.s.	n.s.	n.s.	n.s.	n.s.	n.s.	n.s.	n.s.	n.s.	n.s.

ASC: agroecological service crop; TE: termination; PG: plant growth; EH: end of harvest; CRP: creeping vs. not creeping species; PRN: perennial life form vs. annual; SBL: long-term seed bank longevity vs. short-term; SWT: seed weight; CH: canopy height; SLA: specific leaf area; BFF: beginning of flowering (flowering onset); DFF: duration of flowering (flowering span); L: light affinity; T: temperature; N: soil nutrient condition (N, T and L, Ellenberg and Pignatti values); R: species richness; TD/C: total density for TE and PG stages and total cover for EH one. Probability level: n.s., not significant; *, *p* ≤ 0.05; **, *p* ≤ 0.01; ***, *p* ≤ 0.001.

**Table 4:** Weed traits, Ellenberg ecological indicators, species richness and total density/cover of the weed communities in the two-years experiment as affected by the ASC species.

	CRP	PRN	SBL	SWT	CH	SLA	BFF	DFF	L	T	N	R	TD/C
**TE 2014**	n.s.	n.s.	n.s.	n.s.	n.s.	*	n.s.	n.s.	n.s.	n.s.	n.s.	n.s.	*
**TE 2015**	n.s.	n.s.	n.s.	n.s.	n.s.	n.s.	n.s.	n.s.	n.s.	n.s.	n.s.	*	*
**PG 2014**	n.s.	n.s.	n.s.	n.s.	n.s.	n.s.	n.s.	n.s.	n.s.	n.s	n.s.	*	***
**PG 2015**	n.s.	n.s.	n.s.	n.s.	***	*	*	*	n.s.	*	n.s.	*	*
**EH 2014**	n.s.	n.s.	n.s.	n.s.	n.s.	n.s.	n.s.	n.s.	n.s.	n.s.	n.s.	*	n.s.
**EH 2015**	n.s.	n.s.	n.s.	n.s.	n.s.	n.s.	n.s.	n.s.	n.s.	n.s.	n.s.	n.s.	n.s.

ASC: agroecological service crop; TE: termination; PG: plant growth; EH: end of harvest. ^2^ CRP: creeping vs. not creeping species; PRN: perennial life form vs. annual; SBL: long-term seed bank longevity vs. short-term; SWT: seed weight; SLA: specific leaf area; BFF: beginning of flowering (flowering onset); DFF: duration of flowering (flowering span); L: light affinity; T: temperature; N: soil nutrient condition (N, T and L, Ellenberg and Pignatti values); R: species richness; TD/C: total density for T and PG stages and total cover for EH. Probability level: n.s., not significant; *, *p* ≤ 0.05; **, *p* ≤ 0.01; ***, *p* ≤ 0.001.

## Supplementary Materials

The following are available online at https://www.mdpi.com/2223-7747/9/6/689/s1, Figure S1: Spider graph comparing traits, Ellenberg indicators, weed richness, total density and total cover of the weed communities in ASC presence (ASC yes) and in fallow plots (ASC no) over the two years at TE stage (A), PG stage (B), EH stage (C). CRP: creeping vs. not creeping species; PRN: perennial life form vs. annual; SBL: long-term seed bank longevity vs. short-term; SWT: seed weight; CH: canopy height; SLA: specific leaf area; BFF: beginning of flowering; DFF: duration of flowering (flowering span); L: light affinities; T: temperature; N: soil nutrient condition (N, T and L, Ellenberg and Pignatti values); R: species richness; TD: total density; TC: total cover. Figure S2. Effect of ASC species on SLA (a–b), CH (c), BFF (d), DFF (e) weed traits and T Ellenberg ecological indicator (f) at TE-2014 (a) and PG-2015 stages (b–f).
