# Supplementary material for: Weed Functional Diversity as Affected by Agroecological Service Crops and No-Till in a Mediterranean Organic Vegetable System
Source: Plants (Basel). 2020 May 28;9(6):689. doi: 10.3390/plants9060689 (PMC7356168; doi:10.3390/plants9060689)
Supplement: Supplementary file 1 [file plants-09-00689-s001.pdf]

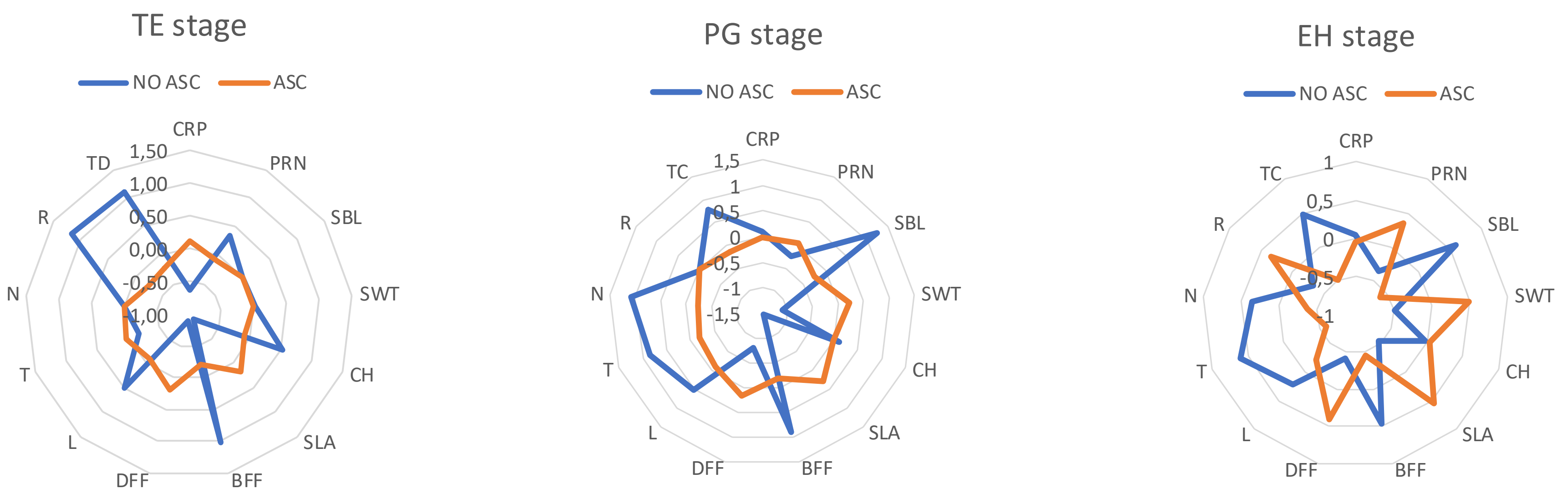

(a) (b) (c)

Figure S1. Spider graph comparing traits, Ellenberg indicators, weed richness, total density and total cover of the weed communities in ASC presence (ASC yes) and in fallow plots (ASC no) over the two years at TE stage (a), PG stage (b), EH stage (c). CRP, creeping vs not creeping species; PRN, perennial life form vs annual; SBL, long-term seed bank longevity vs short-term; SWT, seed weight; CH canopy height; SLA, specific leaf area; BFF, beginning of flowering; DFF, duration of flowering (flowering span); L, light affinity; T, temperature; N, soil nutrient condition (N, T and L, Ellenberg and Pignatti values); R, species richness; TD, total density; TC, total cover.

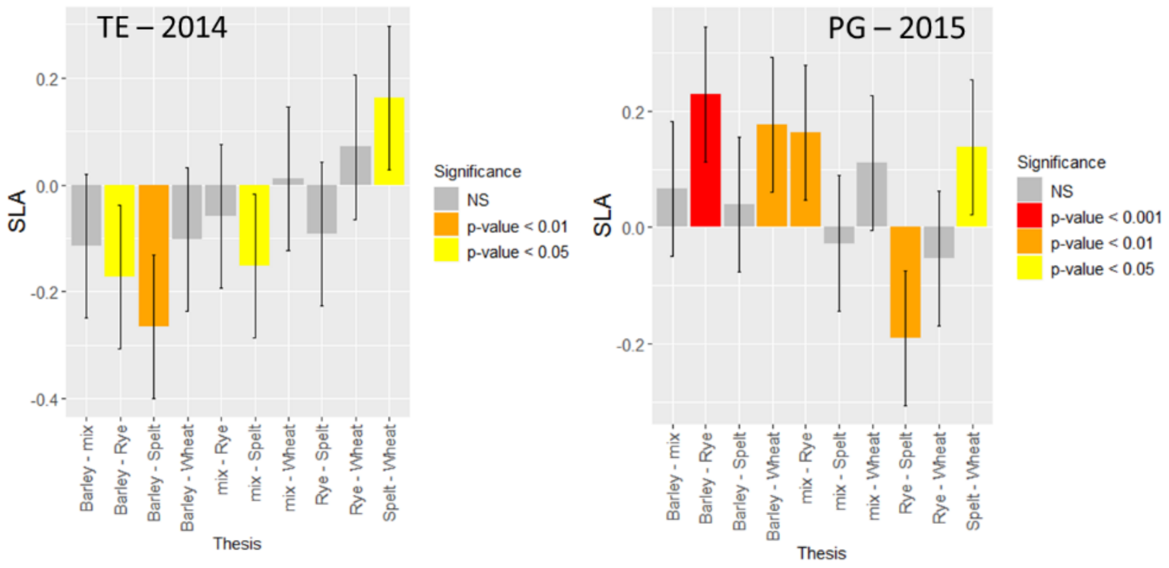
TE – 2014
SLA
Barley - mix
Barley - Rye
Barley - Spelt
Barley - Wheat
mix - Rye
mix - Spelt
mix - Wheat
Rye - Spelt
Rye - Wheat
Spelt - Wheat
Thesis
Significance
NS
p-value < 0.01
p-value < 0.05
PG – 2015
SLA
Significance
NS
p-value < 0.001
p-value < 0.01
p-value < 0.05

(a) (b)

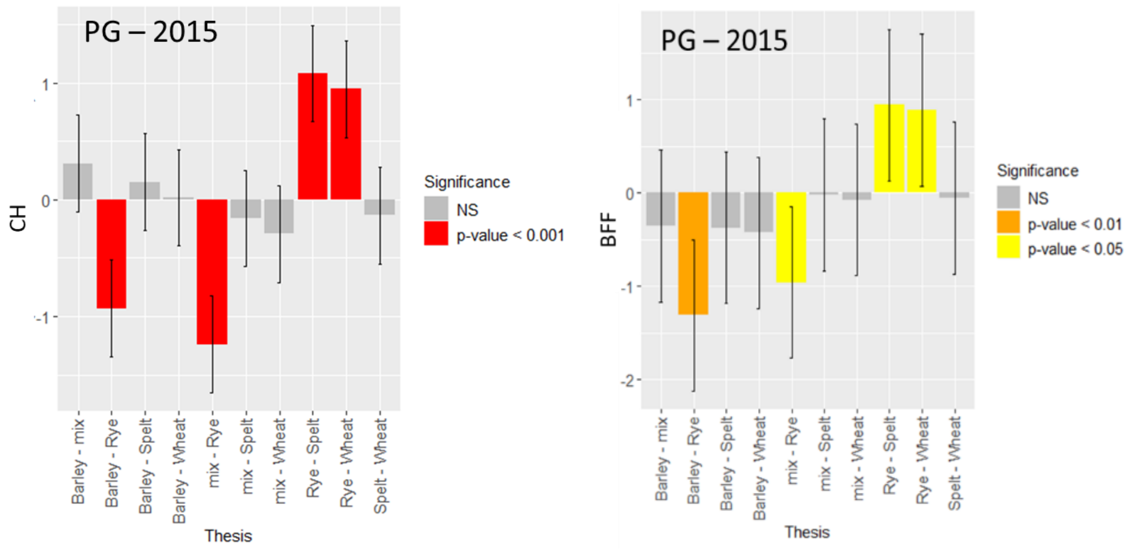
PG – 2015
CH
Thesis
Significance
NS
p-value < 0.001
PG – 2015
BFF
Thesis
Significance
NS
p-value < 0.01
p-value < 0.05

(c) (d)

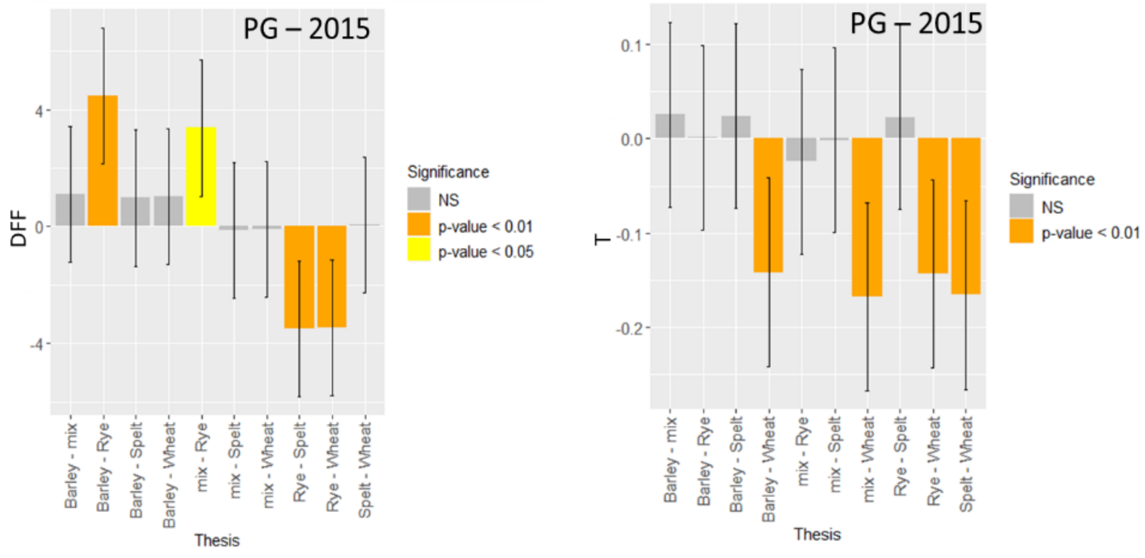
PG – 2015
DFF
Thesis
Significance
NS
p-value < 0.01
p-value < 0.05
PG – 2015
T
Thesis
Significance
NS
p-value < 0.01

(e) (f)

Figure S2. Effect of ASC species on SLA (a-b), CH (c), BFF (d), DFF (e) weed traits and T Ellenberg ecological indicator (f) at TE-2014 (a) and PG-2015 stages (b-f). CH, canopy height; SLA, specific leaf area; BFF, beginning of flowering; DFF, duration of flowering (flowering span); T, Ellenberg indicator for temperature.

NS – not significant difference

© 2020 by the authors. Submitted for possible open access publication under the terms and conditions of the Creative Commons Attribution (CC BY) license (http://creativecommons.org/licenses/by/4.0/).
